# Reflection Losses Analysis from Interspacing between the Cells in a Photovoltaic Module Using Novel Encapsulant Materials and Backsheets

**DOI:** 10.3390/ma12132067

**Published:** 2019-06-27

**Authors:** Asma Shamim, Muhammad Noman, Adnan Daud Khan

**Affiliations:** U.S-Pakistan Center for Advanced Studies in Energy, University of Engineering & Technology, Peshawar 25000, Pakistan

**Keywords:** reflection losses, interspacing, solar cells, PV module, encapsulant, backsheet

## Abstract

Higher efficiency and output power of a photovoltaic (PV) module can be achieved by minimizing cell-to-module (CTM) power losses. CTM losses are mainly dependent on electrical and optical losses. In this work, reflection losses from interspacing of cells with respect to different encapsulant materials and backsheets are evaluated. Two novel encapsulant materials thermoplastic polyolefin (TPO) and polybutadiene ionomer are used, in addition to conventionally used ethylene vinyl acetate (EVA). Moreover, the effect of using these encapsulant materials separately with Tedlar and Aluminum foil as backsheets is realized. It has been observed that TPO in combination with Tedlar presents minimum reflection losses compared to other encapsulant materials. The reflection losses calculated experimentally with polybutadiene ionomer were 5.4% less than the conventionally used EVA, whereas, the reflection losses calculated experimentally with TPO were 5.9% less than the conventionally used EVA. The experimental results obtained are also validated through simulations.

## 1. Introduction

The cell-to-module (CTM) power ratio can be defined as the ratio of module power to cell power, multiplied by the total number of solar cells incorporated in the module. The polycrystalline solar cell efficiency recently launched in the market is about 21%, but when translated into its corresponding module, its efficiency decreases to 19% [1]. To maximize the efficiency of the photovoltaic (PV) module, it is vital to minimize CTM losses as much as possible.

In the last few years, solar cell efficiencies have increased significantly. However, the gain could not always be transferred when processed into PV modules. Apart from recombination, electrical and optical losses are the main factors that limit the performance of PV modules. Electrical losses are usually associated with the metal grid connections, while optical losses are caused by light reflection and absorption in the glass and encapsulant. Researchers are trying hard to mitigate these losses by optimizing the design of solar cells and modules. Moreover, different encapsulant materials and backsheets are being employed to reduce the reflection losses through interspaces between the cells [2,3,4,5]. 

The interspaces between the solar cells in a PV module do not have the ability to absorb the incident photons; hence, they do not contribute to power generation and thus are wasted. However, incident light can be redirected from inactive module areas to solar cells due to lambertian reflection from backsheet. Henceforth, the performance of the PV module can be determined as a function of cell spacing [6,7]. Moreover, absorption of light is strongly dependent on the properties of backsheet as well as the encapsulant material used in the PV module. Therefore, backsheet and the encapsulant material impact the overall performance of the PV module.

Due to far-reaching technological improvement, crystalline Si is considered to be the best understood and most widely used PV material. However, the rapid growth of the PV industry has provided an opportunity for thin films [8,9] and multijunction-based PV modules to enter the market [10,11,12]. Most crystalline Si-based PV modules use a conventional front metal grid to transport power from solar cells to external load. However, it is less known that the presence of these interconnections at the front are prone to both optical and resistive losses that impact the performance and cost of the PV modules. While conventional crystalline Si-based PV technology will make further improvements in cost and performance, it will take a variation in the basic PV architecture to achieve significant improvements.

The back contact solar cells offer a new crystalline Si-based PV architecture that provides the advantage of having more surface area for photon absorption. Moreover, the manufacturing cost of such PV module is half as much as conventionally used front metal grid PV modules [13,14]. The simplification of the module assembly with negligible optical losses is the most revealing benefit of the back contact PV module [15]. A recent study shows that almost 70% of the module cost is analogous to the processing of silicon cells, and most of that cost is associated with patterned metallization [16]. While using back contact configuration, this cost can be reduced significantly as aluminum foil can be used to make electrical connections [17,18,19].

The encapsulation material plays an important role in improving the efficiency and durability of a PV module. Conventionally, ethylene vinyl acetate (EVA) is used as an encapsulant material. However, prolonged UV exposure and extreme weather conditions can cause EVA discoloration, known as the encapsulant browning or yellowing effect [20]. The encapsulant browning can drastically effect the performance and reliability of PV modules [21,22]. To overcome this problem, research on an alternative encapsulant material that is more optically, electrically, and mechanically stable than EVA is still undergoing [23,24].

Thermoplastic polyolefin (TPO), a polymeric blend of thermoplastic polyolefins and olefinic elastomers, is an interesting candidate for PV encapsulation because of its high electrical resistivity and low cost [1,3]. Moreover, it is resistant to hydrolysis and does not degrade under acetic acid formation like EVA, and hence it is less prone to encapsulant browning [1,6]. Polybutadiene ionomer on the other hand also belongs to the category of thermoplastic and are produced from unsaturated carboxylic acid co-monomers and ethylene. In last few years, the UV stability of ionomers has been established in architectural applications [1,15]. Moreover, no formation of acetic acid has been observed during weathering [1]. Compared to EVA, they have more improved moisture sensitivity, lower water vapour transmission rate, higher volume resistance, and a higher degree of mechanical stability [5,7,8]. All these properties make them a potential candidate to be used as an encapsulate material in PV modules.

In this work, reflection losses from the inter spacing of the cells are investigated by using two novel encapsulant materials (TPO and polybutadiene ionomer) that are less prone to encapsulant browning along with traditionally used EVA. Moreover, aluminum foil is used as a backsheet in addition to conventional Tedlar.

## 2. Experimental Methodology

### 2.1. Sample Preparation

**Figure 1:**
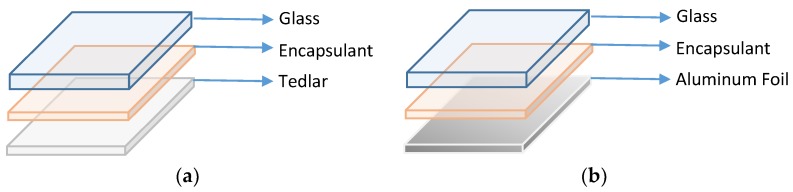
Mini coupon layer orientation with (**a**) Tedlar as back sheet and (**b**) aluminum foil as back sheet.

In this work, samples were prepared by using NPC Photovoltaic Module Laminator LM-110 × 160-S (NPC, Tokyo, Japan). Initially, vacuum pump pressure of 100 KPa was attained an hour before lamination. Two different sets of samples with 5 cm × 2.5 cm dimension were prepared as shown in Figure 1. The first set was comprised of conventional Tedlar as a backsheet, while the second set consisted of cost-effective aluminum foil. Both sets were used in combination with low-iron, non-tempered borosilicate glass as front surface and different encapsulant materials sandwiched between them. Apart from commercially used EVA, two non-conventional encapsulant materials (TPO and polybutadiene ionomer) were used to analyze the impact on reflection losses. By carefully laying up all the layers, as shown in Figure 1, the samples were thermally bonded using 140 °C, 150 °C, and 160 °C for EVA, polybutadiene ionomer, and TPO, respectively, for both sets. Finally, the samples were cooled down on heat-resistant surface for 20 min.

### 2.2. Optical Characterization

**Figure 2:**
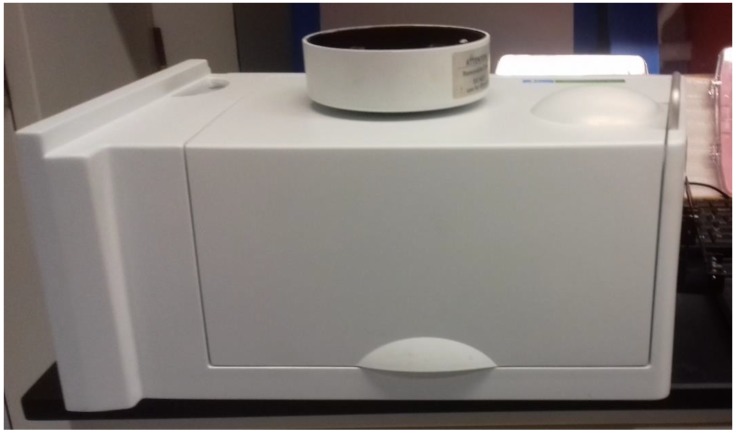
Perkin Elmer’s Lambda 950 UV-VIS-NIR spectrophotometer.

The reflectance in the wavelength range of 300 nm to 1200 nm from the surface of all the samples was measured using Perkin Elmer’s Lambda 950 UV-VIS-NIR spectrophotometer (Perkin Elmer, Waltham, MA, USA), as shown in Figure 2. This apparatus is capable of measuring various optical properties of a sample from ultraviolet to near infrared wavelength range. Before measurements, each sample is cleaned with clean room particles-free wipes that are soaked in ethanol and DI water, respectively.

Each measurement is repeated five times to eradicate the doubts in the physical parameter, and 95% confidence interval mean is reported in the plots. During the experiment, 100% T/0A Baseline and the 0% T/Blocked Beam Baseline readings were taken to eliminate the error of transmittance. Furthermore, to eliminate any doubt in the measurement of reflectance, following equation was used for corrected reflectance:

(1) $$R_{c}=\frac{\left(S_{sample}-S_{dark}\right)}{\left(S_{std}-S_{dark}\right)}\times R_{std}$$

where *R_c_* is the corrected reflectance, *S_sample_* is the spectrophotometer measurement when sample loaded at the port, *S_dark_* is the spectrophotometer signal recorded for the dark current measurements, *S_std_* is the spectrophotometer measurement when standard reflectance loaded at the port, and *R_std_* shows reflection for calibrated reflectance standard.

### 2.3. Simulations

The reflection losses calculated with spectrophotometer were validated using SunSolve, a PV Lighthouse software. Two sets of samples were prepared as mentioned in Figure 1, by varying encapsulant materials. As this research includes the use of two non-conventional encapsulant materials (TPO and polybutadiene ionomer), it is necessary to find out extinction coefficient (k) and refractive index (n) first, as the required simulation data of non-conventional materials are not available in the software. The k and n are calculated using MATLAB, a custom method explained in [25], which requires the reflectance (R_m_) and transmittance (T_m_) values from spectrophotometer. In this method, R_m_ and T_m_ of both TPO and ionomer measured through spectrophotometer subsume in Equations (2) and (3) are simultaneously solved using MATLAB code to get sample interface reflection (R) and fraction of light absorbed per pass (A). The values of R and A are subsequently incorporated in Equations (4) and (5) to acquire refractive index (n) and absorption coefficient (α). Finally, the extinction coefficient (*k*) is calculated by substituting the value of (α) in Equation (6).

(2) $$R_{m}=R\left[\frac{1+\left(1-2R\right){\left(1-A\right)}^{2}}{1-{\left(1-A\right)}^{2}R^{2}}\right]$$

(3) $$T_{m}=\frac{{\left(1-R\right)}^{2}\left(1-A\right)}{1-{\left(1-A\right)}^{2}R^{2}}$$

(4) $$n=\frac{1+\sqrt{R}}{1-\sqrt{R}}$$

(5) $$\alpha =\frac{\ln\left(1-A\right)}{W}$$

(6) $$\alpha \left(\lambda \right)=\frac{4\pi k\left(\lambda \right)}{\lambda }$$

## 3. Results and Discussion

**Figure 3:**
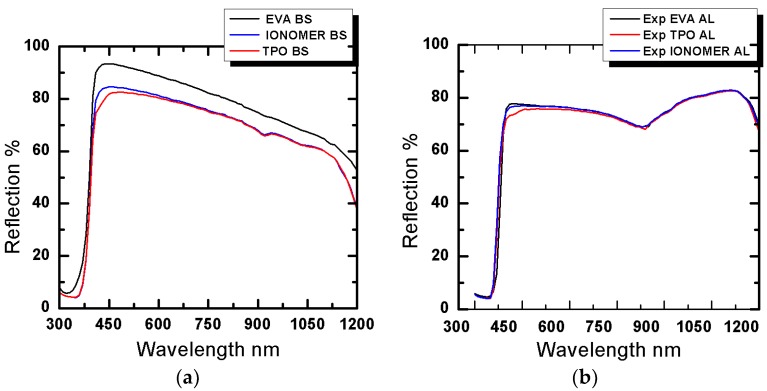
Reflection along the electromagnetic spectrum using different encapsulants for (**a**) samples with Tedlar as backsheet and (**b**) samples with aluminum foil as backsheet.

The reflection losses from two sets of samples were calculated using spectrophotometer over 300 nm to 1200 nm wavelength range, as shown in Figure 3. The first set was comprised of Tedlar as back sheet, while second set consisted of aluminum foil as back contact. It is quite evident from the figure that in case of Tedlar, EVA has more inter spacing reflection losses than polybutadiene ionomer and TPO. Hence, along with the advantage of lower degradation rate, polybutadiene ionomer and TPO have fewer optical losses compared to traditionally used EVA. 

As far as the second set with aluminum foil at back surface is concerned, polybutadiene ionomer and TPO have again proved to have fewer losses compared to EVA. The reflection losses from all the samples are tabulated in Table 1, which clearly depicts that best result can be achieved when TPO is used in combination with Tedlar. It is worth to mention here that the reflection losses with TPO were 5.9% less than the conventionally used EVA when Tedlar was used as a back sheet.

**Figure 4:**
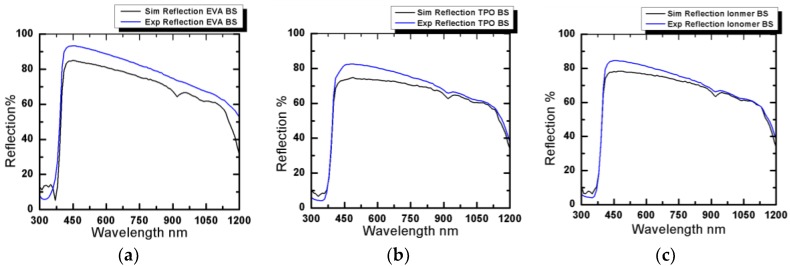
Reflection along the light spectrum using Tedlar as back sheet for (**a**) ethylene vinyl acetate (EVA), (**b**) thermoplastic polyolefin (TPO), and (**c**) polybutadiene ionomer.

**Figure 5:**
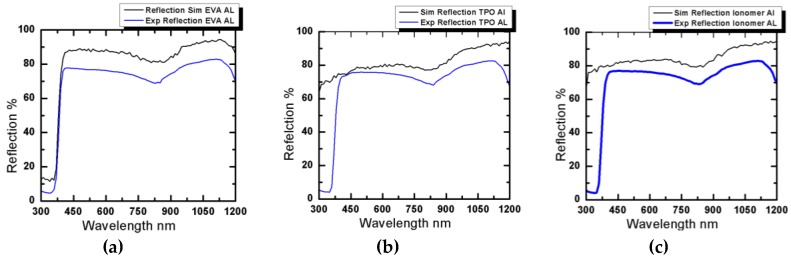
Reflection along the light spectrum using aluminum foil as back sheet for (**a**) EVA, (**b**) TPO, and (**c**) polybutadiene ionomer.

All the experimental results obtained from spectrophotometer are validated through simulation using SunSolve software. Figure 4 presents both experimental and simulation results for reflection losses from inter spacing using Tedlar as back sheet, while Figure 5 compares the experimental and simulation results for reflection losses from inter spacing using aluminum foil as back contact. 

All experimental values had good agreement with simulation results, as shown in Figure 4 and Figure 5. Here, the slight change between the experimental and simulation results are ascribed to experimental errors and sub-micron level flaws inherent in the glass bulk material.

**Figure 6:**
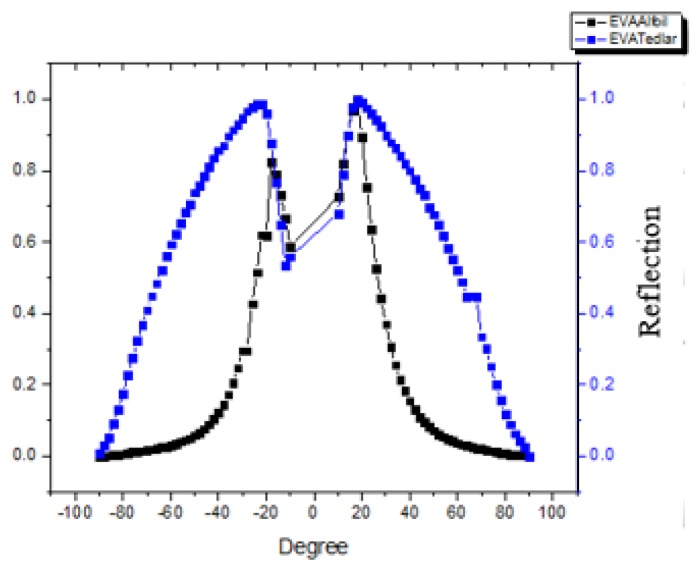
Average reflection from ARTA for both types of module having EVA.

To counter check the results obtained in Table 1, where reflection losses in case of Tedlar are far less than in case of aluminum, a set of experiments was performed using automated reflectance/transmittance analyzer (ARTA) spectrophotometer. The first set consisted of Tedlar with conventional EVA, while the second set consisted of aluminum with conventional EVA. It has been observed that, over the wavelength range of 300 nm–1200 nm, the average total internal reflection from Tedlar is much wider than aluminum, as shown in Figure 6, which shows strong lambertian nature of Tedlar. The lambertian reflection allows the light to scatter at much wider angles from interspacing, and therefore increases the probability of light absorption in the cells significantly. 

## 4. Conclusions

In the work presented in this paper, the investigation of reflection losses from interspaces between the solar cells were realized using mini coupons. Two sets of samples were prepared. One set is comprised of Tedlar as a back sheet, while a second set was made up of aluminum foil as a back contact. Both types of sets were prepared in combination with either EVA, TPO, or polybutadiene ionomer. It has been observed that samples with TPO as an encapsulant and Tedlar as a backsheet present the lowest reflection losses compared to other samples. Therefore, it is concluded that TPO can be a potential candidate as an encapsulant material to replace traditional EVA in the future, as it not only provides the lowest reflection losses from solar cells but also is less prone to encapsulant browning. 

## Figures and Tables

**Table 1:** Reflection losses from interspacing with different encapsulant/backsheet combination using spectrophotometer.

	Tedlar	Aluminum Foil
EVA	54.3%	71.4%
Polybutadiene ionomer	48.81%	71.18%
TPO	48.4%	70.42%
